# Multicellular PID control for robust regulation of biological processes

**DOI:** 10.1098/rsif.2024.0583

**Published:** 2025-01-29

**Authors:** Vittoria Martinelli, Davide Fiore, Davide Salzano, Mario di Bernardo

**Affiliations:** ^1^ Department of Mathematics and Applications, 'R. Caccioppoli' University of Naples Federico II Via Cintia Monte S.Angelo, Naples 80126, Italy; ^2^ SSM- School for Advanced Studies Via Mezzocannone 4, Naples 80138, Italy; ^3^ Department of Electrical Engineering and Information Technology, University of Naples Federico II Via Claudio 21, Naples 80125, Italy

**Keywords:** synthetic biology, biological control, genetic regulatory systems, proportional-integral-derivative control

## Abstract

This article presents the first implementation of a proportional-integral-derivative (PID) biomolecular controller within a consortium of different cell populations, aimed at robust regulation of biological processes. By leveraging the modularity and cooperative dynamics of multiple engineered cell populations, we develop a comprehensive *in silico* analysis of the performance and robustness of P, PD, PI and PID control architectures. Our theoretical findings, validated through *in silico* experiments using the BSim agent-based simulation platform for bacterial populations, demonstrate the robustness and effectiveness of our multicellular PID control strategy. This innovative approach addresses critical limitations in current control methods, offering significant potential for applications in metabolic engineering, therapeutic contexts and industrial biotechnology. Future work will focus on experimental validation *in vivo* and further refinement of the control models.

## 1. Introduction

Synthetic biology is a field that leverages engineering principles to endow cells with new functionalities [1]. By designing innovative genetic circuits for integration into living cells, this field enables organisms to produce valuable chemicals or acquire novel capabilities, such as environmental sensing [2,3]. The applications of synthetic biology are diverse, spanning medicine, environmental science, agriculture and the food industry. In the medical domain, for instance, researchers are developing microbial diagnostics and therapies for diseases that have eluded conventional treatments [4,5]. Furthermore, engineered microorganisms, such as yeasts, are being used to create biofuels as sustainable alternatives to fossil fuels [6]. Additionally, synthetic biology enables the production of innovative food designed to meet specific nutritional requirements [7].

The integration of synthetic gene networks with the native circuits of the host cell, along with the inherently nonlinear and stochastic nature of biochemical processes, can lead to unintended effects. Therefore, there is a pressing need for more reliable strategies to regulate gene expression. These strategies must ensure robust regulation of the output response while preserving the natural adaptive properties of living cells. Specifically, these strategies should maintain the desired steady-state behaviour even under variable conditions [8].

The emerging field of *cybergenetics*, which merges synthetic biology with control theory, presents novel opportunities for robust regulation of biological processes. This interdisciplinary approach involves the design of synthetic feedback control architectures to ensure stable biological functioning [9–11]. High-performance solutions from control theory, such as nonlinear and predictive control strategies, are considered for this purpose. However, the availability of suitable biological components is limited, posing significant constraints on the feasible designs of practical controllers.

In response, the use of proportional-integral-derivative (PID) controllers using biomolecular components has gained traction due to their simplicity in implementation and their ability to provide robust regulation. Specifically, the use of an integral action is able to guarantee precise regulation to a desired set point, as well as robustness to constant output disturbances, a property known as *robust perfect adaptation* [12]. The addition of a proportional action has been shown to enhance the transient performance and reduce the stationary variance across the different cells within the population [13,14]. Finally, by including a derivative term to the control action, it is possible to further suppress fluctuations within the population, as well as enhancing response time and damping of the closed-loop system. This action can be incorporated either by modulating transcription burst rates [15], or designing additional regulatory networks based on integration [16] or incoherent feedforward actions [17]. An alternative route to implement biochemical PID controllers based on chemical reaction networks (CRN) relies on the dual rail formalism, where two species are used to overcome the impossibility of exerting negative control actions. These embedded controllers, which can be realized using DNA displacement reactions [18], have been mathematically shown to improve dynamic performance in a deterministic setting and reduce fluctuations in a stochastic scenario [19]. An extensive review on embedded PID controllers can be found in [20].

Despite the advancements, these solutions assume that all the control functionalities can be implemented within the same cell. However, the integration of all necessary biological components within a single cell introduces challenges such as excessive metabolic burden and reduced modularity. These limitations hinder the broader applicability and scalability of the developed systems, potentially restricting their use in complex applications like immune cell engineering for cancer therapy. Thus, while the literature on embedded biomolecular PID controllers is rich with proposals, practical implementation and scalability remain critical hurdles.

Transitioning from an embedded to a multicellular approach offers a promising strategy to address the limitations associated with engineering all necessary components within one cell. In this multicellular paradigm, different functions are distributed among multiple cell populations within a microbial consortium. This division of labour can significantly minimize metabolic strain and reduce retroactivity—an unintended influence on a system caused by its interaction with another system [21,22].

The physical separation of different biomolecular components across various cell species can also alleviate the metabolic load on individual host cells. This spatial organization allows for more specialized cellular functions, which enhances the overall efficiency and effectiveness of the synthetic system [23].

Examples of successful multicellular control designs have been documented, where engineered microbial communities coordinate their activities through the exchange of signalling molecules. These designs often utilize quorum-sensing molecules to facilitate communication between different cell types, effectively closing the feedback loop necessary for robust system regulation [24,25]. This approach not only leverages the natural communication pathways of microorganisms but also opens new avenues for creating sophisticated, scalable and more naturally integrated synthetic biology applications.

In this article, we introduce a novel multicellular PID controller concept, drawing inspiration from the embedded controller design detailed in Chevalier *et al*. [26], and building on the preliminary work on multicellular PI and PD biomolecular controllers we presented in [27,28]. Our design incorporates orthogonal quorum-sensing molecules to carry out intercellular communication within a microbial consortium.

We begin by developing a mathematical model of this consortium to systematically describe its dynamics and interactions. Furthermore, we explore the impact of varying control gains within the four types of controllers in the PID family: P, PD, PI and PID controllers. To analyse these effects, we employ the method of root contours, a control theoretic multi-parameter analytical technique [29]. This methodology extends the root locus analysis previously performed in Filo *et al*. [20,30], allowing for a more comprehensive understanding of the control dynamics under different gain settings.

Our analysis provides practical guidelines for selecting the appropriate control strategy and tuning the control gains to meet specific performance criteria. To validate our theoretical findings, we conduct *in silico* experiments using BSim [31], an agent-based realistic simulator of cellular populations. These simulations help confirm the effectiveness and robustness of our proposed multicellular PID control approach under various simulated conditions.

## 2. Results

In what follows, we present the development and design of multicellular control architectures implementing a family of PID controllers. We discuss the effects of varying control gains within each controller population and validate our results through *in silico* experiments.

### 2.1. Multicellular proportional-integral-derivative control architectures

We present a set of four multicellular control architectures, each comprising different engineered cell populations that implement the biological equivalents of the classical PID control schemes. Assuming the combination of all control actions, the classical PID control law can be expressed as follows:


(2.1) $$\begin{array}{lcr} & u_{PID}(t):=u_{P}(t)+u_{I}(t)+u_{D}(t)=k_{P}e(t)+k_{I}\int_{0}^{t}e(\tau )d\tau +k_{D}\dot{e}(t), & \end{array}$$

where $k_{P}$, $k_{I}$ and $k_{D}$ are the so-called proportional, integral and derivative control gains, respectively, and


(2.2) $$e(t):=y_{d}(t)-y(t)$$

is the control error, that is, the difference between the measured output y(t) of the process to be controlled and its desired value $y_{d}(t)$. The control law in (2.1) is broadly and successfully used in industrial applications due to its simplicity and ease of tunability (see for example, the reference textbook [32]). Indeed, well-assessed techniques exist to select the values for the control gains to meet specific performance requirements.

The control law consists of three terms. The proportional term provides an action proportional to the error e(t), causing it to decrease. However, this alone is insufficient to reduce the error exactly to zero. In theory, the error approaches zero as the proportional gain $k_{P}$ grows larger and larger, but this is not feasible in practice. To this aim, the integral action is used to ensure robust regulation to zero, meaning that the error converges to zero regardless of constant disturbances and parametric uncertainties affecting the controlled process. In other terms, the integral action provides an input that compensates for constant disturbances and reference signals by integrating the error e(t). This ensures that the control signal $u_{I}(t)$ settles to a constant steady-state value only when the error becomes zero. Finally, by using the time derivative of the control error $\dot{e}(t)$, the derivative action is used to make the response faster and to enhance the stability of the closed-loop system.

**Figure 1.**
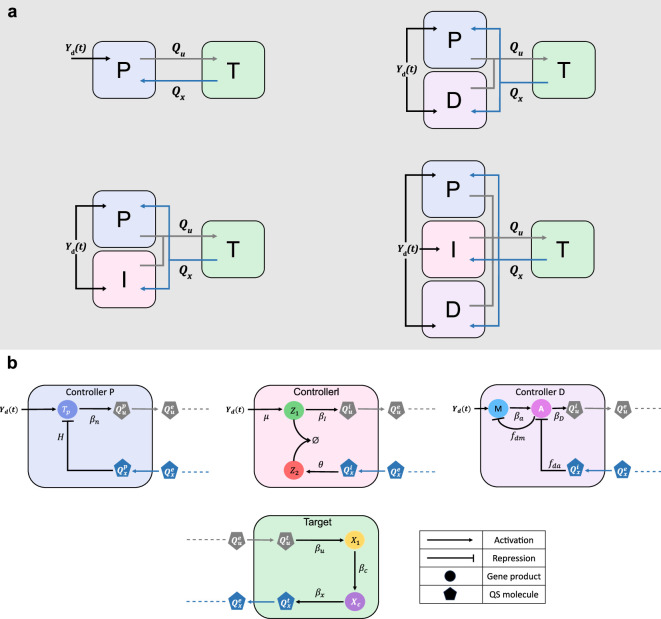
Multicellular PID control architectures. The proposed multicellular control strategy consists of three controller populations that can be combined to realize one of the four multicellular PID control architectures. The molecular communication realized by the two diffusive signals $Q_{u}$ and $Q_{x}$ is crucial to the correct functioning of the consortium for the regulation of the biological process Φ(t) inside the target cells to the desired value $Y_{d}$. The control input depends on the mismatch between the desired output $Y_{d}$ and the measured output $Q_{x}$ produced by the target population that carries the information about the state of the process Φ(t) to the controller populations. (*a*) Schematic representation of the multicellular P, PD, PI and PID control architectures. Different multicellular PID family's controllers can be obtained by combining the three controller populations. (*b*) Abstract biological representation of the proportional (blue), integral (pink) and derivative (purple) controller cells and of the target cells (green). Each type of controller cell embeds a synthetic gene network producing the control molecule $Q_{u}$ according to the corresponding control action. For the sake of simplicity, we assume that the process Φ(t) in the target cells is a genetic network consisting of only two genes, $X_{1}$ and $X_{c}$, where $X_{1}$ is directly actuated by the control signal carried by the control molecule $Q_{u}$, and $X_{c}$ represents the output of Φ(t), which in turn activates the production of the quorum-sensing molecule $Q_{x}$. Circles depict internal molecular species, while polygons represent the quorum-sensing molecules. The superscripts p, i, d and t are used to designate quantities associated with the proportional, integral, derivative and target cells, respectively, and the superscript e to denote quantities into the external environment. The physical meaning of all variables and parameters is reported in table 1. Details on H, $\beta_{n}$, $f_{da}$, $f_{dm}$ are reported in the electronic supplementary material, §S3, equations (S16), (S17) and §S1, equation (S7), respectively.

In our multicellular design, we distribute the PID control actions among three *controller populations*. These cell populations can be combined in various ways to achieve the desired overall control signal that regulates the expression of a biological process Φ(t), such as a target gene, embedded in another *target population*. The modularity of our design allows for different combinations of the controller populations, enabling the realization of all possible architectures within the PID controllers’ family, namely the P, PD, PI and PID controllers, as depicted in figure 1*a*.

**Table 1.** Table of biomolecular species’ symbols and definitions.

symbol	physical meaning
$X_{1},X_{c},Z_{1},Z_{2},A,M$	cells’ internal species
$Q_{k}^{j},Q_{k}^{e}$	internal and external quorum-sensing molecules
$\beta_{u},\beta_{c},\beta_{x},\mu ,\theta ,\beta_{a},\beta_{m}$	activation rates
γ	degradation rate in all cells of the consortium
$\gamma_{a},\gamma_{m}$	active degradation rates of the derivative species
$\gamma_{z}$	rate constant of the annihilation reaction
$\gamma_{e}$	external degradation rate of molecules
$\beta_{P},\beta_{I},\beta_{D}$	activation rates of the control molecule in the controller cells
η	molecules diffusion rate
N	number of cells in each population
$Y_{d}$	reference signal

The exchange of information between the members of the consortium, enabling the cell populations to cooperate, is implemented through a pair of orthogonal quorum-sensing molecules, $Q_{u}$ and $Q_{x}$ [33]. These molecules diffuse into the environment to respectively broadcast the actuation and sensing signals, thereby closing the feedback loop. Specifically, the controllers can sense the mismatch between the reference signal $y_{d}(t)$ and the measured target output y(t), which is broadcast to them through the sensor molecule $Q_{x}$. Based on this information, they produce the control molecule $Q_{u}$ according to their designed control function.

Hence, following the principles of classical control theory, our aim is to regulate the output of the process Φ(t) to a desired value, ensuring that the control error e(t) converges to zero as time approaches infinity. Additionally, we require robust regulation, meaning that parametric uncertainties do not cause excessive deviation from the desired value, and that the transient response is both fast and predictable.

To assess the static and dynamic performance of the controller, we will use the following metrics: the error at steady state, $e_{\infty }=\lim_{t\to \infty }|e(t)|$, for static performance; and the settling time, $t_{s_{\chi \%}}$, that specifies how fast the output y(t) converges inside a bound of χ% around the steady-state value $y_{\infty }=\lim_{t\to \infty }\;y(t)$, and the overshoot, $o_{\%}$, that quantifies how large is the peak value of the oscillations in the response with respect to the steady-state value $y_{\infty }$, for dynamic performance.

However, due to the inherent uncertainties affecting biological systems, as noise and parametric perturbations, asymptotic regulation, i.e. $e_{\infty }=0$, is generally not feasible. Therefore, it is more reasonable to require only bounded regulation, meaning the error norm converges below some prescribed value ε.

Thus, the control objective can be specified as that of achieving:

1. $\lim_{t\to \infty }|e(t)|\leq \varepsilon$

2. $t_{s_{\chi \%}}\leq \tau$

3. $o_{\%}\leq \varpi$

where ε, τ and ϖ are positive values to be specified.

Despite the simplicity of the PID control law and the extensive literature and tools available for tuning its control gains in industrial applications, a systematic and straightforward approach to meet the aforementioned control specifications does not exist in the biological context we are considering here. Moreover, the multicellular architecture adds further complexity to the problem, as it requires the tuning of parameters governing the production and diffusion of signalling molecules between the cell populations. Also, while the classical PID controller family is linear, the biomolecular implementation described in this article is inherently nonlinear making the gain tuning problem particularly cumbersome.

### 2.2. The mathematical model of the multicellular proportional-integral-derivative controllers’ family

Next, we present a mathematical model capturing the aggregate dynamics of the most comprehensive architecture, which includes all the controller populations in the consortium, namely the multicellular PID controller. This aggregate model describes the average behaviour and interactions of the biochemical species within the microbial consortium, allowing us to analyse the system’s overall performance. The mathematical models for the other control architectures depicted in figure 1*a*, i.e. P, PD and PI controllers, can be directly obtained from the model of the PID controller by setting the corresponding control gains to zero.

In order to derive the aggregate dynamics, we make several simplifying assumptions. Specifically, we assume that all populations within the consortium grow and divide at the same rate. This implies that each species is also diluted at the same rate, say γ. This is a reasonable assumption when all engineered populations use the same microbial host. Furthermore, we assume that all populations maintain an equal number of cells, denoted by N, ensuring that the consortium populations are balanced in size. Building on these assumptions, it follows that the quorum-sensing molecules involved in the system have uniform diffusion rates among the populations, denoted by η. For a comprehensive derivation of the agent-based and aggregate mathematical models, please refer to the electronic supplementary material, §§S1 and S2.

In what follows, we use superscripts p, i, d and t to designate quantities associated with the proportional, integral, derivative and target cells, respectively, and the superscript e to denote quantities into the external environment.

First, we provide the dynamics of the species produced in the target cells, which constitute the process to control, namely Φ(t). This process can be modelled as a network of different chemical species $X_{1},\ldots ,X_{c}$, with $X_{1}$ being the input species and $X_{c}$ the output species. For simplicity, we model the process in its simplest form, as done in [12], where the expression of $X_{c}$ is directly influenced by $X_{1}$. The abstract biological scheme of the network within the target cells is depicted in figure 1*b*, where the quantity $Q_{u}^{t}$ represents the actuation signal to the target cell, and the molecule $Q_{x}^{t}$ represents its measured output. Using mass-action kinetics, the dynamics of the species produced into the targets can be written as


(2.3) $$\begin{array}{r} {\dot{X}}_{1}=\beta_{u}Q_{u}^{t}-\gamma X_{1}, \end{array}$$

(2.4) $$\begin{array}{r} {\dot{X}}_{c}=\beta_{c}X_{1}-\gamma X_{c}, \end{array}$$

(2.5) $$\begin{array}{r} {\dot{Q}}_{x}^{t}=\beta_{x}X_{c}+\eta (Q_{x}^{e}-Q_{x}^{t})-\gamma Q_{x}^{t}. \end{array}$$

The meaning of all variables and parameters is provided in table 1.

Next, we describe the dynamics of the control populations, whose schematic biological representation is shown in figure 1*b*, each producing a contribution to the control quorum-sensing molecule $Q_{u}$. Specifically, the dynamics of the proportional cells can be reduced to the following equation:


(2.6) $${\dot{Q}}_{u}^{p}=\beta_{P}Y_{d}\frac{\mu Y_{d}}{\mu Y_{d}+\theta Q_{x}^{p}}+\eta (Q_{u}^{e}-Q_{u}^{p})-\gamma Q_{u}^{p},$$

where $\beta_{P}$ represents the maximal production rate of the control molecule and serves as a proportional gain.

The second contribution to $Q_{u}$ is provided by the integral cells, whose aggregate dynamics can be written as


(2.7) $$\begin{array}{r} {\dot{Z}}_{1}=\mu Y_{d}-\gamma_{z}Z_{1}Z_{2}, \end{array}$$

(2.8) $$\begin{array}{r} {\dot{Z}}_{2}=\theta Q_{x}^{i}-\gamma_{z}Z_{1}Z_{2}, \end{array}$$

(2.9) $$\begin{array}{r} {\dot{Q}}_{u}^{i}=\beta_{I}Z_{1}+\eta (Q_{u}^{e}-Q_{u}^{i})-\gamma Q_{u}^{i}, \end{array}$$

where $\beta_{I}$ plays the role of an integral control gain. Here, we assumed the annihilation reaction between $Z_{1}$ and $Z_{2}$ to be their only source of degradation, as done in previous works [26,34]. However, dilution of these species should be accounted for in a more realistic scenario, as done in [35].

The final contribution to the control molecule $Q_{u}$ is produced by the gene network embedded within the derivative cells, which can be modelled using Michaelis–Menten kinetics, leading to the following equations:


(2.10) $$\begin{array}{r} \dot{A}=\beta_{a}M-\gamma_{a}Q_{x}^{d}\frac{A}{K_{a}+A}-\gamma A, \end{array}$$

(2.11) $$\begin{array}{r} \dot{M}=\beta_{m}Y_{d}-\gamma_{m}A\frac{M}{K_{m}+M}, \end{array}$$

(2.12) $$\begin{array}{r} {\dot{Q}}_{u}^{d}=\beta_{D}A+\eta (Q_{u}^{e}-Q_{u}^{d})-\gamma Q_{u}^{d}, \end{array}$$

where $\beta_{D}$ plays the role of a derivative control gain.

Finally, we model the dynamics of the molecules diffusing into the cells where they are not produced as


(2.13) $$\begin{array}{r} {\dot{Q}}_{u}^{t}=\eta (Q_{u}^{e}-Q_{u}^{t})-\gamma Q_{u}^{t}, \end{array}$$

(2.14) $$\begin{array}{r} {\dot{Q}}_{x}^{j}=\eta (Q_{x}^{e}-Q_{x}^{j})-\gamma Q_{x}^{j}, \end{array}$$

where j∈{p,i,d}, and their dynamics in the external environment as


(2.15) $${\dot{Q}}_{k}^{e}=\eta N\sum_{j\in S}(Q_{k}^{j}-Q_{k}^{e})-\gamma_{e}Q_{k}^{e},$$

where k∈{x,u} and S={t,p,i,d}.

The mathematical model described by equations (2.3)–(2.15) can be simplified by making the realistic simplifying assumptions A3–A6 on the system’s timescales (see electronic supplementary material, §S4). This allows us to derive a *reduced model* through timescale separation, thus obtaining


(2.16) $$\begin{array}{r} {\dot{X}}_{1}=\beta_{u}Q_{u}-\gamma X_{1}, \end{array}$$

(2.17) $$\begin{array}{r} {\dot{X}}_{c}=\beta_{c}X_{1}-\gamma X_{c}, \end{array}$$

(2.18) $$\begin{array}{r} \dot{\zeta }=\mu Y_{d}-\theta Q_{x}, \end{array}$$

where $\zeta =Z_{1}-Z_{2}$. Here, the steady-state values of the quorum-sensing molecules and the species A, respectively, are defined as


(2.19) $$\begin{array}{r} Q_{x}=\frac{1}{\Gamma_{PID}}\beta_{x}X_{c}, \end{array}$$

(2.20) $$\begin{array}{r} Q_{u}=\frac{1}{\Gamma_{PID}}\left(\beta_{P}Y_{d}\frac{\mu Y_{d}}{\mu Y_{d}+\theta Q_{x}}+\beta_{I}\zeta +\beta_{D}A\right), \end{array}$$

(2.21) $$\begin{array}{r} A=-\frac{1}{\Gamma_{PID}}\frac{\gamma_{a}\beta_{x}}{\beta_{a}\gamma_{m}}{\dot{X}}_{c}+\frac{\beta_{m}}{\gamma_{m}}Y_{d}, \end{array}$$

where $\Gamma_{PID}=4\gamma$. Further details on the derivation of the reduced order model can be found in the electronic supplementary material, §S4. Starting from the reduced model with PID control, (2.16)–(2.18), it is possible to derive the models of the other multicellular control strategies by setting the gains of the controller populations that are not present in the consortium to zero (i.e. $\beta_{I}=\beta_{D}=0$ for the P controller, $\beta_{I}=0$ for the PD controller and $\beta_{D}=0$ for the PI controller). Additionally, equation (2.18) is removed when the integral cells are not present in the architecture, as is the case for the P and PD multicellular controllers.

### 2.3. Effects of tuning control gains in multicellular control architectures

PID controllers have a different effect on the output response depending on the control actions used and the chosen control gains. Therefore, selecting the appropriate controller is crucial to achieve the desired system performance. However, so far, differently from industrial applications, well-established tools to straightforwardly select the right values for the control gains given the desired static and transient responses are not available for the biological architecture proposed here.

Therefore, our goal is to provide guidelines for selecting the appropriate control strategy and gains to meet specific control requirements. As a result of our work, we derive analytical conditions for guiding the consortium design by evaluating the steady-state performance and the transient characteristics of the output response using multicellular P, PD, PI and PID control architectures.

Despite the reduced model (equations (2.16)–(2.18)) simplifying the analysis of the closed-loop system dynamics, the equations remain highly nonlinear, making it difficult to evaluate how the parameters affect performance. Therefore, we performed a local analysis by linearizing the reduced model around the desired set point, computing the closed-loop transfer function in the Laplace domain, and finally evaluating the effect of changing the control gains on the closed-loop response (see §S5 of the electronic supplementary material for more details on the derivation). As it will be shown, this approach reveals clear relationships between control gains and closed-loop performance, which, however, hold only locally to the desired operating point and not globally. This limitation does not undermine the significance of our results, as synthetic biology applications are typically designed to operate near nominal conditions, that is, to have desired performance around a set point, and characterize local properties through linearization about that point [36,37]. Moreover, the validity of the control architectures we propose here will also be extensively tested in non-local conditions through *in silico* experiments.

The transfer function of a linear dynamical system completely describes the relationship between the input and output signals, illustrating how the closed-loop system behaves when a prescribed signal, such as the reference signal $y_{d}(t)$, is applied to it. The transfer function of the closed-loop system regulated by the complete PID controller can be expressed as follows:


(2.22) $$\begin{array}{lcr} & G_{cc,PID}(s)=\frac{\theta Q_{x}(s)}{\mu Y_{d}(s)}=\frac{\left(3\kappa_{P}s+\kappa_{I}+\frac{\beta_{a}\beta_{m}\theta }{\mu \gamma_{a}}\kappa_{D}s\right)\theta \beta_{c}}{s^{3}+(2\gamma +\theta \beta_{c}\kappa_{D})s^{2}+(\gamma^{2}+\theta \beta_{c}\kappa_{P})s+\theta \beta_{c}\kappa_{I}}, & \end{array}$$

where $Q_{x}(s)$ and $Y_{d}(s)$ are the Laplace transform of the time-dependent signals $Q_{x}(t)$ and $Y_{d}(t)$, and s is the complex variable. Here, the control gains $\kappa_{P}$, $\kappa_{I}$ and $\kappa_{D}$ come from aggregations of the system parameters, and are defined as


(2.23) $$\kappa_{P}:=\frac{\beta_{P}\beta_{u}\beta_{x}}{4\Gamma_{\ell }^{2}\mu },\;\ell \in \{P,PD,PI,PID\},$$

(2.24) $$\kappa_{I}:=\frac{\beta_{I}\beta_{u}\beta_{x}}{\Gamma_{\ell }^{2}},\;\ell \in \{PI,PID\},$$

(2.25) $$\kappa_{D}:=\frac{\beta_{D}\beta_{u}\beta_{x}\gamma_{a}}{\Gamma_{\ell }^{2}\beta_{a}\gamma_{m}\theta },\;\ell \in \{PD,PID\}.$$

Additionally, $\Gamma_{\ell }=M\gamma$ for M∈{2,3,4}, where M is the number of populations in the consortium. Note that the transfer functions for other control schemes, such as the P, PD and PI control schemes shown in figure 1*a*, can be derived from equation (2.22) by setting the corresponding control gains to zero. For example, by setting $\kappa_{I}=0$ and $\kappa_{D}=0$ for the P control scheme.

Next, we assessed the local performance of each control architecture leveraging the static gain of the transfer function (2.22) and its poles by means of the root contours analysis [29] detailed in electronic supplementary material, §S6.

#### 2.3.1. Robust asymptotic regulation can be guaranteed only by integral action

Asymptotic regulation, meaning that the control error e(t), defined as the difference between the constant desired value $y_{d}=\mu Y_{d}$ and the measured output $y(t)=\theta Q_{x}(t)$, converges to zero as t goes to infinity, is achieved when the static gain of the closed-loop transfer function in equation (2.22) is unitary.

The static gain is formally defined as the limit of the transfer function (2.22) as s tends to 0. In general, it may depend on both the control gains and the parameters of the biological system. For example, for the P controller the static gain can be computed from (2.22) to be $\frac{3\theta \beta_{c}\kappa_{P}}{\gamma^{2}+\theta \beta_{c}\kappa_{P}}$. However, when the integral controller is used, i.e. $\kappa_{I}\neq 0$, the static gain is always equal to 1, making it independent of the system parameters.

This means that the integral control action, similar to the classical control law, guarantees robust asymptotic regulation. Consequently, constant perturbations from the nominal value of the parameters do not affect the output value at steady state.

Although the static gain can be equal to 1 also without the integral controller, i.e. $\kappa_{I}=0$, as in the P and PD architectures, the choice of the control gains $\kappa_{P}$ and $\kappa_{D}$ that guarantee this condition depends strictly on the nominal value of the parameters of the biological system. Indeed, for the static gain to be unitary, the two control gains must satisfy the following condition:


(2.26) $$\begin{array}{lcr} & \kappa_{D}+\kappa_{P}\frac{2\gamma_{a}\mu }{\beta_{a}\beta_{m}\theta }-\frac{\gamma^{2}\gamma_{a}\mu }{\beta_{c}\beta_{a}\beta_{m}\theta^{2}}=0. & \end{array}$$

This dependence is a mathematical identity; therefore, in the P and PD cases, regulation is not robust with respect to parametric uncertainties, which are unavoidable in real biological systems. However, unlike the use of P or PD controllers, where the stability of the closed-loop system is always preserved, using an integral controller requires careful tuning of the control gains to ensure stability. Specifically, for the PI or PID control architectures, a unique equilibrium point exists when the control gains are adequately tuned to satisfy the condition


(2.27) $$\kappa_{D}+\kappa_{P}\frac{2\gamma_{a}\mu }{\beta_{a}\beta_{m}\theta }-\frac{\gamma^{2}\gamma_{a}\mu }{\beta_{c}\beta_{a}\beta_{m}\theta^{2}}\leq 0.$$

Such an equilibrium is locally exponentially stable when


(2.28) $$\begin{array}{lcr} & \kappa_{I}<\left(\frac{2\gamma +\kappa_{D}}{\theta \beta_{c}}\right)(\gamma^{2}+\kappa_{P}\theta \beta_{c}). & \end{array}$$

From equation (2.28), it can be observed that, compared with the PI controller (where $\kappa_{D}=0$), the PID controller extends the range of $\kappa_{I}$ values for which the equilibrium remains asymptotically stable. This indicates that the derivative action has a stabilizing effect on the closed-loop system.

**Figure 2.**
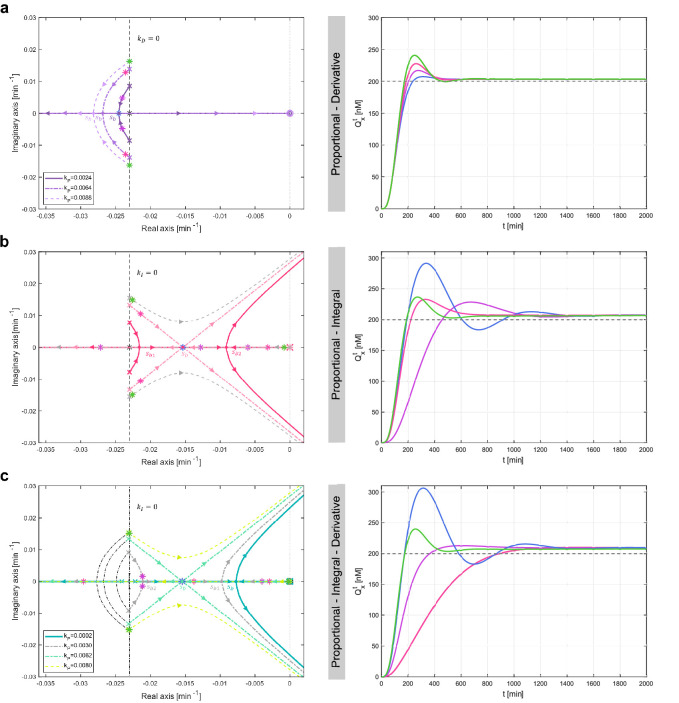
Characterization of the proposed multicellular PID family control architectures. Root contours (left panels) and time evolution of the concentration of the measured output $Q_{x}^{t}$ corresponding to fixed values of the control gains (right panels) when the targets are controlled by the PD, PI and PID controllers, respectively; the P controller being a special case of the PD controller when $\kappa_{D}$ is set to zero. The starting points of the root contours of the PD and PI architectures, respectively, lie on the root locus of $\kappa_{P}G_{P}(s)$, computed from equation (2.22) when only $\kappa_{P}\neq 0$, whereas the root contours of the PID control architecture all start on the root contours of $\kappa_{D}G_{PD}(s)$, computed from equation (2.22) when $\kappa_{P},\kappa_{D}\neq 0$. Fixing the value of $\kappa_{P}$ for the PD and PI architectures, and of $\kappa_{P}$ and $\kappa_{D}$ for the PID architecture, allows to pick out one of the root contours. Next, changing the value of $\kappa_{D}$ or $\kappa_{I}$, respectively, places the closed-loop poles at a specific location along the selected root contour, affecting the closed-loop transient and steady-state responses. (*a*) Effects of changing the derivative gain on the closed-loop poles. We selected some values of $\kappa_{D}$ meeting the condition (2.26) when only the proportional and derivative cells are included into the consortium. First, we considered $\kappa_{D}=0$ (green star), that is, the proportional controller alone. Next, we set $\kappa_{D}\neq 0$ (pink, purple and blue stars, respectively) to observe the effects of adding a derivative action on the closed-loop response. Specifically, the two real coincident poles indicated with the blue star were chosen by selecting $\kappa_{D}$ meeting also condition (S79) to obtain the fastest possible response. (*b*) Effects of the PI controller on the closed-loop poles. We selected different values of the integral gain $\kappa_{I}$ fulfilling conditions (2.27) and (2.28) (pink, purple, blue and green stars, respectively). Specifically, the three coincident poles indicated with the blue star were chosen to also meet condition (S90) in order to guarantee the fastest possible output response. (*c*) Effects of the PID controller on the closed-loop poles. After fixing admissible values of the proportional and derivative gains (see condition (2.27)), we set some values of $\kappa_{I}$ fulfilling condition (2.28) (pink, purple, blue and green stars, respectively). Specifically, the blue star marks the three coincident real poles that are placed by choosing $\kappa_{I}$ meeting also the condition derived in §S6.4 to obtain the fastest possible response. The other biochemical parameters were selected as in electronic supplementary material, table S1, whereas the reference signal was set to $Y_{d}=60\;\mathrm{nM}$.

The simulations reported in the right panels in figure 2 demonstrate that when the control gains are tuned according to the aforementioned conditions, resulting in a unitary static gain, the control error at steady state of the complete model (2.3)–(2.15) remains consistent. Moreover, as will be discussed in the next section, the additional degrees of freedom in choosing the control gains for the PD, PI and PID schemes can be further exploited to modify the transient response. This allows us to meet the control specifications for settling time $t_{s_{\chi \%}}$ and overshoot $o_{\%}$.

**Figure 3.**
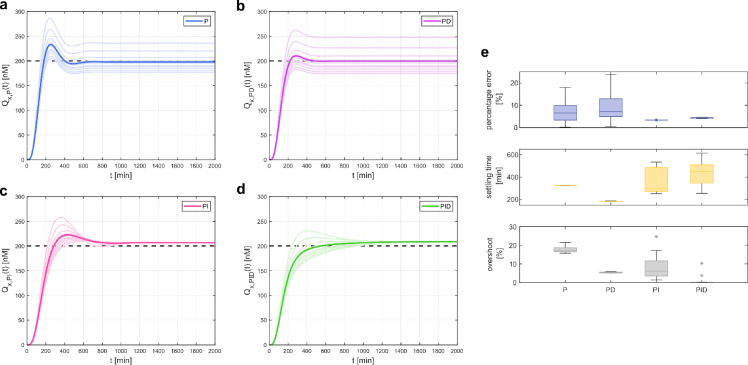
*In silico* regulation experiments in Matlab. The regulation capability of the four control architectures was tested by running *in silico* experiments in Matlab. The control gains have been tuned according to equation (2.27) and from the regions shown in figure 2, fulfilling the control requirements on the transient response, and their values have been kept the same for all architecture to exploit its modularity. For each experiment, the parameters of the target cells were perturbed to test the robustness of the control architecture with respect to uncertainties affecting the biological process Φ(t). Specifically, for each of the 10 simulations performed, the targets' parameters were drawn from a normal distribution centred at their nominal value $\hat{\rho }$ and with standard deviation $\sigma =0.1\cdot \hat{\rho }$. All the other parameters are fixed to their nominal values as in electronic supplementary material, table S1. All the control architectures can drive the error signal near to zero, but only when the integral controller cells are used the steady-state error is independent from perturbation on the parameters of the target cells. (*a–d*) Time evolution of the concentration of the measured output $Q_{x}^{t}$ (lighter lines) when the targets are controlled by P, PD, PI and PID control actions, respectively, as the parameters of the target cells are varied. Darker lines represent the mean of these signals. (*e*) Box plots of the percentage steady-state error (blue), settling time at 10% (yellow) and percentage overshoot (grey) computed at each experiment of the P, PD, PI and PID control strategy, respectively. The central solid lines are the medians; the whiskers have endpoints corresponding to the minimum and maximum value excluding the outliers; the stars represent outliers. The total number of cells for each control strategy is fixed to 120, equally divided between the different cell types. Additionally, we fixed the initial concentrations of all species in the simulations to zero, assuming that each cellular population was grown separately from the others before starting the experiment.

Next, we evaluated the sensitivity of the P, PD, PI and PID control strategies to variations in target parameters to account for parametric uncertainty in biological systems. To exploit the modularity of the proposed multicellular architecture, the values selected for each control population have been consistently used across the different schemes using the same population. Specifically, the same values of control gain $\kappa_{P}$ have been used in the simulations for the P, PD, PI and PID control architectures; the same values of $\kappa_{D}$ have been used for the PD and PID schemes; and the same values of $\kappa_{I}$ have been used for the PI and PID schemes. On average, all the control architectures are able to drive the error signal near zero. However, only by including the integral controller cells in the consortium does the steady-state error become independent of changes in $\kappa_{P}$ and $\kappa_{D}$ and from perturbations in the target parameters, as long as (2.28) is satisfied. These results are shown in figure 3*a–d*, where we performed 10 simulations for each control architecture, drawing the target parameters from a normal distribution centred at their nominal value $\hat{\rho }$ with a standard deviation of $\sigma =0.1\cdot \hat{\rho }$. This analysis was confirmed by the summary statistics shown in figure 3*e*.

#### 2.3.2. Derivative action makes the transient response faster

The properties of the transient response, i.e. how the output reaches its steady-state value, are closely related to the position of the closed-loop poles of the transfer function in equation (2.22) in the complex plane. These poles, which are the roots of its denominator, can be either real (s=a) or complex conjugate pairs (s=a±jb, where j is the imaginary unit). Real poles are associated with aperiodic movement, meaning a response that reaches steady state without oscillations, while complex conjugate poles cause oscillations. The farther away the poles are from the imaginary axis, i.e. the larger the magnitude |a| of their real parts, the quicker their contribution to the output reaches steady state. Therefore, the dynamical response is ‘dominated’ by the slower poles, known as *dominant poles*, which also define the settling time of the output response, $t_{s_{\chi \%}}=\ln\;\chi_{\%}/|a|$. Conversely, the imaginary part of a complex conjugate pair of poles determines the period of the oscillations, and its relation with the real part determines the damping of the oscillations, and hence the maximum overshoot. Specifically, the damping ratio is defined as $\xi =-a/\sqrt{a^{2}+b^{2}}$; the larger the damping, the smaller the overshoot $o_{\%}$.

The position of the poles of the closed-loop transfer function (2.22), and in particular that of the dominant poles, can be changed by varying the control gains $\kappa_{P}$, $\kappa_{I}$ and $\kappa_{D}$. The relationship between the control gains and the position of the poles can be studied by means of the root contours method (further details in §S6 of the electronic supplementary material), which allows to obtain analytical conditions on the control gains to meet the desired control specifications. Namely, the specification on the settling time $t_{s_{\chi \%}}$ translates into requiring that the real part of the dominant poles is smaller than a prescribed value, while the specification on the overshoot $o_{\%}$ translates into requiring the dominant poles to be inside a triangular sector (see further details in electronic supplementary material, §S6). We found that the proportional controller alone is not sufficient to make the response faster. Indeed, the real part of the dominant poles does not depend on $\kappa_{P}$ as they move in the complex plane on the vertical line with coordinate $s_{b_{P}}=-\gamma$ (see figure 2*a*, dashed black line), where γ is related to the growth rate of the cells. Therefore, we need to introduce other controller populations to improve the system response.

Adding a derivative population in the consortium to realize a multicellular PD controller, places the minimum real part achievable by the dominant pole to $s_{b_{PD}}=-\sqrt{\gamma^{2}+\theta \beta_{c}\kappa_{P}}<-\gamma =s_{b_{P}}$, at the same time, reducing the overshoot as $\kappa_{D}$ increases, as also shown in figure 2*a*. Thus, the PD controller can make the transient response faster and more damped than the proportional controller alone for any value of $\kappa_{P}>0$.

In contrast, when integral controller cells are added to the consortium, that is, using the PI and PID control architectures, the settling time worsens since all the branches of the loci are attracted toward the right-hand side. Therefore, the smallest value of the real part of the dominant poles is always greater than $s_{b_{P}}$ (see figure 2*b–c*).

However, adding the derivative action to the PI controller improves the settling time. Indeed for any $\kappa_{D}>0$ it holds that $s_{b_{PID}}=-\frac{2}{3}\gamma -\frac{1}{3}\theta \beta_{c}\kappa_{D}<-\frac{2}{3}\gamma =s_{b_{PI}}$.

In addition, for both the PI and PID control strategies, speeding up the response increases the percentage overshoot. This creates a trade-off between achieving a shorter settling time and minimizing the overshoot.

### 2.4. *In silico* experiments confirm the analysis

In this section, we present *in silico* experiments to assess the effectiveness of the proposed strategies in more realistic conditions and explore the sensitivity to parameter perturbations, and the robustness to imbalances in the composition of the consortium, of the multicellular P, PD, PI and PID control architectures.

The *in silico* experiments were performed in Matlab or BSim, an agent-based simulation platform designed to realistically simulate bacterial population growth dynamics [31]. By carrying out agent-based simulations in BSim, we accounted for cell geometry, cell growth and division, cell-to-cell mechanics and diffusion of molecules; additionally, we defined constraints on the host chamber.

Specifically, in all BSim simulations, we assumed the cells growing in a rescaled version of the microfluidic device used in Shannon *et al*. [38] and in Salzano *et al*. [39], setting a BSim chamber of dimensions 23 × 15 × 1 μm that can host around 120 cells, thus, minimizing the computational time without losing statistical significance. In our numerical experiments using BSim, we simulated the behaviour of individual cells with a deterministic model. This approach was chosen because the computational resources required for an exhaustive set of numerical experiments using more accurate stochastic simulations would have been beyond our reach, making the analysis unfeasible.

For the *in silico* experimental campaign conducted here, we require the output response of the target cells to converge at steady state to the desired value with an error less than 10%, i.e. $e_{\infty }\leq 0.1$, to account for the effect of parametric disturbances affecting biological systems. Additionally, we require the transient response to settle in less than 24 h, that is, $t_{s_{10\%}}\leq 1440\;\min$, which is a typical timescale in controlled experimental environments [40]. Furthermore, oscillations, if present, must vanish at steady state, and the overshoot should not exceed 20% of the desired steady-state value, i.e. $o_{\%}\leq 20\%$. Oscillations and excessive overshoots are undesirable in many industrial and pharmaceutical applications, such as when engineering T-cells for cancer treatment [41]. In all simulations, we selected the control gains according to the conditions derived in §2.3 and the regions depicted in figure 2 that, for each control strategy, fulfil the prescribed control requirements.

To emphasize the modularity of the proposed control strategies, we consistently used the same values of the control gains in all control architectures, as already done in §2.3.1. We started assessing the performance of the architecture in a stochastic scenario by conducting agent-based simulations in Matlab, where the evolution of the concentration of each chemical species within the cells was simulated using a stochastic simulation algorithm (SSA) [42]. To reduce the computational burden of the simulations, here we neglected growth and movement of cells, as well as the spatial effects related to the diffusion of quorum sensing molecules in the chamber. The architecture successfully regulated $Q_{x}$ to the desired value even in the presence of stochasticity, with performances that are comparable to the ones obtained in a deterministic setting shown in figure 3. Specifically, as can be observed in electronic supplementary material, figure S3, in the presence of noise there was no statistically significant change on the steady-state error, as confirmed by pairwise *t*‐test performed on deterministic and stochastic error distributions. Also, although we observed a general degradation of the transient performance in terms of settling time and percentage overshoot when biochemical noise was present, the relative performance of the different controllers of the family did not change.

Although some studies suggest that the use of a biomolecular derivative action can mitigate noise effects [15], our numerical observations (see electronic supplementary material, figure S4) did not show any significant improvement in noise attenuation at steady state when the derivative action was applied. This might indicate that our proposed implementation of the derivative action does not effectively reduce biochemical noise. Exploring alternative biomolecular implementations of the derivative action that can also enhance noise mitigation is an interesting research topic; however, this falls outside the scope of our present work, which is primarily focused on regulating the average population-level response.

**Figure 4.**
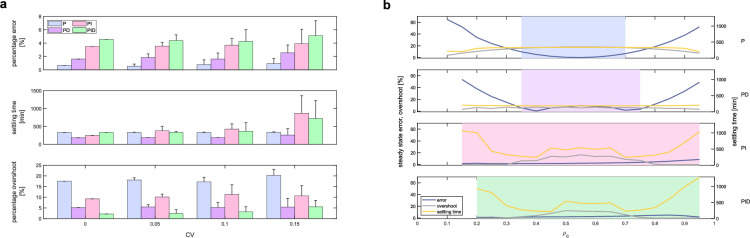
Robustness of the control architectures to parameter variations and population imbalances in BSim. All control architectures showed consistent performance as the uncertainty in the value of the biological parameters increases. Moreover, it appears that when the integral action is used in the consortium, the regulation capabilities of the multicellular architecture becomes more robust to imbalance between the relative number of controller and target cells. (*a*) Percentage error at steady state, settling time and overshoot as parameter variations increase when the targets are controlled by a P (blue bars), a PD (purple bars), a PI (pink bars) and PID (green bars) controller, respectively. For each value of the CV∈{0.05,0.1,0.15} we performed n=20 experiments, drawing the activation parameters of the controllers and the targets from a normal distribution centred at their nominal value $\hat{\rho }$ and with standard deviation $\sigma =CV\cdot \hat{\rho }$. The dimensions of the BSim microfluidic chamber were different for each control strategy, in order to obtain approximately the same number of target cells at steady state for all the control architectures. The metrics were computed filtering the average targets output signal with a moving average filter of window width W=60. The initial number of cells for each control strategy was equally divided between the different cell types. (*b*) Steady-state percentage error, settling time and overshoot of the P, PD, PI and PID control architectures as the percentage of the controller cells, $\rho_{c}:=N_{c}/N$, is varied, with N being the total number of cells in the chamber. All the simulations were performed in BSim setting the growth rate to zero in a chamber of dimensions 23 × 15 × 1 μm hosting in total N=20 cells. The shaded areas highlight the values of $\rho_{c}$ corresponding to successful simulations, in which $e_{\infty }\leq 0.1$, with a settling time $t_{s,10\%}\leq 1440\;\min$ and oscillations disappear at steady state with an overshoot $o_{\%}\leq 20\%$. In all experiments, the reference signal is fixed to $Y_{d}=60\;nM$ , while the gains are chosen as $\beta_{P}=0.0414$, $\beta_{D}=0.0933$ and $\beta_{I}=0.0002\;min^{-1}$. The BSim growth and mechanical parameters were selected as in Fiore et al. [24], while the nominal biochemical parameters were chosen as in electronic supplementary material, table S1.

Next, to evaluate the sensitivity of the system, that is, its robustness to cell-to-cell parameter variations, which naturally exist even between cells of the same species, we ran several simulations in BSim. Each time a cell splits to form two daughter cells, we assigned different values to the targets’ and controllers’ parameters. Specifically, we drew the parameters from a normal distribution centred at their nominal value $\hat{\rho }$ with a standard deviation $\sigma =CV\cdot \hat{\rho }$, where CV is the coefficient of variation. The collected data showed that all the control architectures exhibit consistent performance as the intensity of the perturbation increases (figure 4*a*). Specifically, the steady-state error remained almost the same as CV increased for all the strategies, fulfilling the desired requirement, i.e. $e_{\infty }\leq 0.1$. Regarding the settling time, the P and PD controllers showed similar performance for any of the values of the CV we considered, while the PI and PID controllers exhibited different behaviours as parameter perturbations increased. For CV=0.15, the settling time of the PI controller increased by approximately 2.5 times compared with when there were no perturbations (CV=0), while the PID controller’s settling time increased by approximately 1.2 times. Despite these variations, both strategies generally met the required condition $t_{s_{10\%}}\leq 1440\;\min$, although some simulations of the PI controller exceeded this limit.

Finally, the percentage overshoot $o_{\%}$ remained below 20% as CV increased for all control strategies, except for the P controller when CV=0.15, where it slightly exceeded this upper bound. These data are reported in figure 4*a*, where the relative percentage steady-state error is defined as


(2.29) $$\begin{array}{lcr} & e_{\%}=\frac{1}{n}\sum_{k=1}^{n}\left|\frac{{\hat{Q}}_{x,k}^{t}-Q_{d}}{Q_{d}}\right|\times 100\%, & \end{array}$$

with ${\hat{Q}}_{x,k}^{t}$ being the value of $Q_{x,k}^{t}(t)$, averaged over the last 600 min, of the kth experiment, $Q_{d}=\frac{\mu Y_{d}}{\theta }$ the desired value of $Q_{x}$ and n the total number of experiments for each value of CV.

Next, we considered the presence of imbalances in the consortium composition, which might arise from the different metabolic loads associated with the expression of each control action, affecting the growth rate of the cells.

To test robustness to imbalances, we performed several simulations in BSim, each with a different relative number of cells across the populations while neglecting cell growth and division, and assuming the same number of cells in each controller population. The assumption of neglecting cell growth was done to reduce the computational burden of the simulations, reducing dramatically the execution time, thus allowing the investigation of a larger set of conditions. The presence of the integral cells in the consortium provides robustness (shaded areas in figure 4*b*), and ensures also a smaller error at steady state. However, the settling time $t_{s_{10\%}}$ worsens for the PI and PID controllers, although it remains under 1440min. Meanwhile, the percentage overshoot $o_{\%}$ stayed almost constant and below 20% for all the control strategies. We also extended the analysis to account for imbalances due to each control population in all the control schemes. Specifically, by varying the percentage of one controller at a time, we investigated the effects that different compositions have on the steady-state error, obtaining similar results (see the electronic supplementary material, figure S5).

For each value of $\rho_{c}$ the steady-state error has been evaluated as follows:


(2.30) $$\begin{array}{lcr} & \bar{e}=\frac{1}{t_{f}-t_{s}}\int_{t_{s}}^{t_{f}}e(\tau )d\tau , & \end{array}$$

where $e(t)=\mu Y_{d}-\theta Q_{x}^{t}$ is the error signal, $t_{s}=1440\;\min$ and $t_{f}$ is the simulation time.

## 3. Discussion

In this article, we introduced a novel multicellular PID control strategy for regulating biological processes. Our comprehensive mathematical analysis and model derivation together with *in silico* experiments demonstrate the robustness and efficacy of this approach, highlighting several key findings and potential applications, as well as areas for future improvement.

Our work successfully demonstrated a modular design approach, where PID control actions are distributed among three distinct controller populations within a consortium. This modularity allows for flexible combinations to achieve desired control architectures (P, PD, PI and PID) tailored to specific biological processes. The mathematical models developed for these control architectures, derived from the aggregate dynamics of the system, enable systematic analysis of their performance. This flexibility is crucial for tailoring the control strategies to various biological contexts, ensuring precise and effective regulation.

We found that, despite the nonlinear nature of the biomolecular implementation we presented, the inclusion of integral controller cells in the consortium guarantees robust asymptotic regulation as is the case in classical PID controllers. This robustness is evident as the steady-state error remains independent of parametric variations, provided that specific conditions are met. Such robust regulation is essential for maintaining system stability despite the inherent parametric uncertainties in biological systems. This result is particularly relevant for applications where precise control is paramount, such as the development of reliable devices and networks in synthetic biology and metabolic engineering.

Furthermore, our analysis showed that adding derivative action to the control strategy significantly enhances the transient response by reducing the settling time and dampening oscillations. This improvement in transient performance is critical for applications requiring quick and stable responses. However, it is also noteworthy that while the PI and PID control architectures improve steady-state accuracy, they introduce trade-offs in transient performance, particularly in terms of increased settling time. These trade-offs must be carefully managed to balance the overall system performance.

The *in silico* experiments conducted using the BSim platform validated our analytical results in the presence of more realistic effects including cell growth, division and molecular diffusion.

Our multicellular PID control strategy has broad potential in applications. In synthetic biology, it can be applied to various scenarios where precise control of gene expression is essential, such as in metabolic engineering for optimizing the production of pharmaceuticals or biofuels. In biomedical engineering, our approach can be extended to therapeutic contexts, such as engineering T-cells for cancer treatment, where maintaining a stable and precise response to environmental cues is crucial for effective therapy. Additionally, in industrial biotechnology, where microbial consortia are used for chemical production or bioremediation, our control strategy can enhance process stability and yield by mitigating the effects of environmental and parametric uncertainties.

Despite the promising results, there are areas for future improvement. A key challenge towards the experimental validation of the proposed strategy is guaranteeing the long-term coexistence between the different populations within the consortium. To this aim, it is necessary to develop additional embedded circuits or external controllers that shape the composition of the consortium. In this context, we are exploring the use of multi-chamber bioreactors that could be used to grow different cell populations in different chambers and combine them in a mixing chamber where densities could be carefully controlled using strategies such the ratiometric control techniques presented in [39,43]. Additionally, although we propose a plausible abstract biological implementation of the design, a careful selection of components is required to implement all the proposed functionalities. For example, the integral control action can be realized using cognate σ anti-σ pairs, as done in [34,44]. Instead, as illustrated previously in [26], the proportional control action can be implemented using an inducible transcription factor, whose activity can be modulated by the concentration of the reference signal, as done in [45,46]. Finally, a derivative action can be implemented using the two-node circuit proposed in [47] or one of the other circuits presented in [48].

Further additional work could focus on developing more sophisticated models that capture additional layers of biological complexity, such as stochastic effects, spatial heterogeneity and dynamic interactions among cell populations. Implementing advanced optimization algorithms for tuning control gains could further improve the performance and robustness of the control architectures, making them more adaptive to varying biological conditions. Exploring the scalability of our control strategies for larger and more complex consortia, as well as their integration with other synthetic biology tools and frameworks, will be crucial for broader application and adoption.

## Data Availability

The code used for all simulations is available at [49]. Supplementary material is available online [50].
